# Cardiovascular disease risk factors in homeless people

**DOI:** 10.3109/03009734.2011.586737

**Published:** 2011-06-29

**Authors:** Margit Kaldmäe, Mihkel Zilmer, Margus Viigimaa, Galina Zemtsovskaja, Karel Tomberg, Tanel Kaart, Margus Annuk

**Affiliations:** ^1^Tallinn University, Institute of Mathematics and Natural Sciences, Tallinn, Estonia; ^2^Tartu University, Department of Biochemistry, Tartu, Estonia; ^3^North Estonia Regional Hospital, Tallinn, Estonia; ^4^Estonian University of Life Sciences, Institute of Veterinary Medicine and Animal Sciences, Tartu, Estonia; ^5^Egeen International Corporation, Mountain View, USA

**Keywords:** Atherosclerosis, cardiac diseases, inflammation, pathology

## Abstract

**Background:**

Cardiovascular diseases (CVD) are associated with significant morbidity and mortality, which is highest in Eastern Europe including Estonia. Accumulating evidence suggests that life-style is associated with the development of CVD. The aim of this study was to evaluate the informative power of common CVD-related markers under unhealthy conditions.

**Subjects:**

Subjects (*n* = 51; mean age 45 years; 90% men) were recruited from a shelter for homeless people in Tallinn, Estonia, and consisted of persons who constantly used alcohol or surrogates, smoked, and were in a bad physical condition (amputated toes, necrotic ulcers, etc.).

**Methods:**

Blood pressure, pulse rate, and waist circumference were measured, and body mass index (BMI) was calculated. The following markers were measured in blood serum: total cholesterol (TChol), high-density lipoprotein cholesterol (HDL-Chol), low-density lipoprotein cholesterol (LDL-Chol), plasma triglycerides (TG), apolipoproteins A-l (ApoA1) and B (ApoB), lipoprotein(a) (Lp(a)), glycated hemoglobin (HbA1c), glucose (Gluc), high-sensitivity C-reactive protein (hsCRP), serum carbohydrate-deficient transferrin (CDT), gamma-glutamyltransferase (GGT), alanine aminotransferase (ALT), and aspartate aminotransferase (AST). Except smoking, the anamnestic information considering eating habits, declared alcohol consumption and medication intake were not included in the analysis due to the low credibility of self-reported data.

**Results:**

More than half of the investigated patients had values of measured markers (hsCRP, TChol, LDL-Chol, TG, HbA1c, ApoA1, ApoB, Lp(a), Gluc) within normal range. Surprisingly, 100% of subjects had HDL-Chol within endemic norm.

**Conclusion:**

This study demonstrates that traditional markers, commonly used for prediction and diagnosis and treatment of CVD, are not always applicable to homeless people, apparently due to their aberrant life-style.

## Introduction

Cardiovascular diseases (CVD) are one of the most common clinical diagnoses in the world associated with significant morbidity and mortality, which is highest in Eastern Europe including Estonia (1–3). Also compared to international standards CVD readings (risk factors) in Estonia are considered to be higher (4,5).

A large body of experimental data and results from cross-sectional studies indicate relationships between CVD and total cholesterol (TChol), low-density lipoprotein cholesterol (LDL-Chol), high-density lipoprotein cholesterol (HDL-Chol), high-sensitivity C-reactive protein (hsCRP), plasma triglycerides (TG), and glucose (Gluc) tolerance (6–8). These markers are automatically used to predict, diagnose, and estimate the effectiveness of the CVD treatment but still leave atherosclerosis-related diseases a major challenge for scientists and cardiologists.

CVD is also considered to be the leading cause of death among people who live unsalubrious lives—homeless people (9,10). Undoubtedly, there are factors which are influenced by people themselves, e.g. unhealthy diet, physical inactivity, smoking, and alcohol abuse (11), but at the same time controversial connections have been demonstrated between life-style and CVD risk factors (12–14).

Considering Estonia, homelessness became apparent in the mid-1990s (15), but the data concerning the number of homeless people are questionable. The main reasons for becoming homeless in Estonia are unemployment (85%) and/or alcoholism (60%) (16). Most of homeless individuals have health problems, but to keep their self-esteem the self-estimation of the health condition is generally considered not bad (17). In addition there are so far no studies carried out in Estonia describing the health problems specific to homeless people from a clinical point of view nor their morbidity pattern and death rates.

Through investigating homeless people (persons who live unsalubrious lives and are commonly ‘CVD-labeled’), the aim of this study was to assess traditional CVD-related markers under unhealthy conditions and to evaluate the actual informative power of traditional risk markers as risk factors or predictive markers for CVD.

## Subjects

The study was approved by the Tallinn Medical Research Ethics Committee, and written informed consent was obtained from all of the participants (46 males and 5 females, mean age 45 ± 12.5 years, range 19–66 years). The recruitment and the procedures (measurements, blood sample drawing, etc.) took place in the District Shelter (situated in Mustamäe Tallinn, Estonia) which is a place where homeless people can stay for the night (when not alcohol-intoxicated) but no systematic food provision, medication, or any other services are rendered.

A homeless person was considered eligible for the investigation if the following criteria were met:

- did not have permanent job
- did not have a regular income
- did not have a permanent home
- constantly used alcohol or surrogates
- did not have systematic (regular) eating habits
- was not engaged in regular physical activity.

The pre-recruitment procedure consisted of selection of the asocial contingent, based on the knowledge of the shelter staff, who excluded those subjects (7 out of 58) whom they met for the first time or had seen rarely. All the others (*n* = 51) were confirmed as eligible for the study, based on the above-mentioned criteria. The selection, with assistance from the staff, was followed by administration of a questionnaire consisting of questions about education, work, life-style (how many years of being homeless, eating habits, smoking, drugs and alcohol consumption (what and how much and for how long time), physical activity (how long walks and how many hours outside)) and medical background (what illnesses and what kind of treatment).

The self-reported data mentioned below, based on participants' answers, were not included (except smoking—94% of participants confirmed to be smokers) in the analysis, due to low credibility, which was found through many reasons: when specific information met a contradiction with the data given by the staff of the shelter, subjects denied or seemed to be ashamed of their condition, were doubtful, did not remember. Still the questionnaire gave us the extra proof that they really were suitable for the study considering our criteria.

The mean time of homeless status was approximately 4 years (3.6 ± 3.2 years). Even if the visible physical condition spoke for itself, half of the participants denied or had a doubt of having any illness, pain, or trauma. The other half of the participants mostly claimed to have head, back, or leg traumas, but pneumonia, arthritis, and asthma were also mentioned—25% used painkillers but the specific necessary treatment or medication was still not obtained. Also, for the purpose of finding out the participants' medical background, a search of the medical data was performed, which did not give any significant results.

All participants confirmed using alcohol or surrogates almost every day but did not give an overview specifically what, in what amount, and for how long periods of time they had been consuming.

Even though they all said that they had approximately two meals a day, it could not be counted as systematic eating due to the fact that no food was served at the shelter and also the information given by the participants was doubtful (subjectivity arose as to what is meant by the term ‘a meal’).

Contradictions were also found concerning information about physical activity—participants claimed to walk approximately 11 km (10.8 ± 7.1) a day, but seeing their physical conditions this was largely questionable.

## Methods

Blood pressure (BP) and pulse rate were measured by using a sphygmomanometer in a sitting position after 5 minutes of rest. In addition to weight and height information, waist circumference was also registered (measuring tape positioned mid-way between the top of the hip-bone and the bottom of the rib-cage). Body mass index (BMI) was calculated as the weight in kilos divided by the square of the height in meters.

### Blood sample collection and transport

All blood samples were obtained in a sitting position. Guidelines, indicating the importance of fasting from food before blood sampling, were given to each participant before the testing period.

After an overnight fast, blood samples were collected between 8.00 and 11.00 a.m., with standard method from vena cubitalis using Vacutainer collection tubes (BD Vacutainer, Belliver Industrial Estate, Plymouth; Becton, Dickinson and Co., UK) as follows: for Gluc determination with preservatives (fluoride oxalate); for glycated hemoglobin (HbA1c) with ethylenediamine tetra-acetic acid (EDTA); for alanine aminotransferase (ALT), aspartate aminotransferase (AST), gamma-glutamyltransferase (GGT), hsCRP, CDT%, TChol, HDL-Chol, LDL-Chol, and TG with clot activator without any more additives or preservatives. Obtained samples were stored without centrifugation and immediately transported at room temperature to the laboratory. For apolipoprotein A-1 (ApoA1), apolipoprotein B (ApoB), and lipoprotein(a) (Lp(a)) determinations whole blood was collected in EDTA collection tubes and centrifuged within 1 hour to obtain plasma, which was subsequently stored and transported on ice to the laboratory.

Other laboratory samples, needed for serum separation, were centrifuged and kept at +4°C till assessed. All determinations were performed by following the standard procedures—on the day of collection and within 12 hours.

### Laboratory methods

Markers from blood serum were determined by using different methods as follows. ALT and AST: reaction rate assessment based on the conversion of NADH to nicotinamide adenine dinucleotide (NAD); GGT: kinetic method based on gamma-glutamyl group transference to glycylglycine; Gluc:enzymatic reference method with hexokinase; hsCRP levels: particle-enhanced immunoturbidimetric method; TChol: cholesterol oxidase technique; HDL-Chol and LDL-Chol: homogeneous enzymatic colorimetric assay; TG: glycerol phosphate oxidase technique after enzymatic cleavage of fatty acids. All of these determinations were performed by reagents from Roche (Roche Diagnostics, Mannheim, Germany) using Roche Integra 800 analyzer.

Blood HbA1c was determined on Roche Integra 400 analyzer using the particle-enhanced immunoturbidimetric method. Final results were expressed as percent HbA1c from total Hb according to Diabetes Control and Complications Trial/National Glycohemoglobin Standardization Program (DCCT/NGSP) protocol. CDT measurement was performed by latex-enhanced reagent from Dade Behring using Behring BN analyzer (Siemens Healthcare Diagnostics, Deerfield, USA).

Apolipoproteins ApoA1 and ApoB were analyzed by using immunoturbidimetric assays and Lp(a) by using particle-enhanced immunoturbidimetric assays (Roche Diagnostics, Mannheim, Germany). The intensity of the turbidity, proportional to the concentration of antibody–antigen complexes, was measured by the Roche Integra 400 analyzer.

### Statistical analysis

Statistical analyses were performed using MS Excel and Statistical package R. The characteristics of studied persons were presented as mean ± standard deviation. 95% confidence intervals of percentages were evaluated with the exact binomial test in R.

## Results

The comparison of data based on mean values versus the reference values (RF) would not give an adequate overview because the single extreme values would significantly skew the average results. Considering this kind of subject distribution (how they fitted within the RF), percentage evaluation in addition to average values was chosen.

During the examination most of the subjects were or seemed to be in a bad physical condition—amputated toes, necrotic ulcers, and other leg and back traumas. Still considering the aim of the study (the scheme was not to analyze their physical state and medical condition) the true condition stayed unknown.

Data in brackets are represented as follows: mean ± SD, range. For indices where reference values are different between male (M) and female (F) study participants the mean ± SD and range values are indicated separately. As the exact values of Lp(a) <0.08 were not known the median value instead of the mean value was calculated.

Approximately a quarter of homeless subjects' BMI values met World Health Organization (WHO) criteria for being overweight (RF ≥25). The rest of the participants (73%) had normal BMI value (22.9 ± 3.0, 17.8–32.7 kg/m²). Also the waist circumference was in normal range among 88.2% of the study persons (M 84.7 ± 10.1, 64.0–110.0 cm; F 95.6 ± 10.5, 86.0–111.0 cm).

Evaluated on the basis of a mean systolic blood pressure (SBP) RF ≥140 mmHg or a mean diastolic blood pressure (DBP) ≥90 mmHg (18), 51.0% of homeless subjects had high blood pressure (SBP 143.4 ± 24.9, 98.0–196.0 mmHg; DBP 82.3 ± 15.2, 52.0–118.0 mmHg).

Pulse rate was also high (>75 b/min) in 68.6% of the persons studied (84.2 ± 14.3, 55.0–112.0 b/min)—16% of them had tachycardia (>100 b/min).

More than half of the patients had hepatic toxification markers in the low or within the normal range: GGT 68.6% (M 85.8 ± 147.9, 12.0–888.0 U/L; F 185.8 ± 268.8, 7.0–646.0 U/L), ALT 70.6% (M 45.7 ± 48.7, 9.0–249.0 U/L; F 38.8 ± 27.4, 12.0–75.0 U/L), AST 51.0% (M 53.1 ± 45.8, 18.0–245.0 U/L; F 47.4 ± 27.2, 17.0–74.0 U/L); and also alcohol abuse marker, CDT 56.9% (2.9 ± 2.1, 0.9–9.0%).

Also the markers which indicate high risk for CVD were in the normal range among more than half of patients: hsCRP 58.8% (12.1 ± 25.3, 0.3–129.0 mg/L), TChol 64.7% (5.0 ± 1.4, 2.9–11.0 mmol/L), LDL-Chol 68.6% (2.7 ± 1.1, 1.3–7.0 mmol/L), TG 88.2% (1.1 ± 0.6, 0.4–4.0 mmol/L), HbA1c 88.2% (5.5 ± 0.3, 4.7–6.5%), ApoA1 84.3% (M 1.7 ± 0.3, 1.2–2.5 g/L; F 1.8 ± 0.8, 1.3–3.1 g/L), ApoB 92.2% (M 0.7 ± 0.2, 0.4–1.3 g/L; F 1.1 ± 0.3, 0.6–1.5 g/L), Lp(a) 56.9% (M 0.08, <0.08–0.9 g/L; F <0.08,0.08–0.2 g/L), Gluc 60.8% (5.4 ± 0.9, 3.7–8.7 mmol/L).

When looking at the mean values of these markers, only the hsCRP value was not within the endemic norm. Surprisingly, 100% of the subjects had a normal HDL-Chol value(1.9 ± 0.7, 1.0–4.0 mmol/L). A total of 90.2% of patients had low TChol/HDL ratio (M 2.7 ± 0.8, 1.4–5.0; F 4.3 ± 1.7, 2.6–6.6), and the LDL/HDL ratio was in the normal range among 72.5% of patients (M 1.5 ± 0.7, 0.4–3.6; F 2.7 ± 1.4, 1.3–4.5).

There were no subjects who had all measured markers in the endemic norm.

## Discussion

On the basis of a large body of evidence, TChol, LDL-Chol, HDL-Chol, and TG are automatically used in worldwide clinical practice as the first selection to predict the risk of myocardial infarction (MI) and also to estimate CVD treatment effectiveness.

Interestingly our study consisting of persons who live unsalubrious life showed no significant differences concerning average values of TChol, HDL-Chol, LDL-Chol, and TG compared to reference (endemic norm) values. Also, the percentage distribution demonstrated that significantly more than half of persons studied had these markers in the normal range (Table I).

**Table I.** Characteristics compared to reference values of the study subjects.

Characteristics	No.	%	95% CI
Blood pressure (mmHg)			
Normal (SBP <120 and DBP <80)	8	15.7	(7.5–29.1)
Pre-hypertension (SBP 120–139 or DBP 80–89)	16	31.4	(19.5–46.0)
Stage 1 hypertension (SBP 140–159 or DBP 90–99)	15	29.4	(17.9–44.0)
Stage 2 hypertension (SBP ≥160 or DBP ≥100)	11	21.6	(11.8–35.7)
Not measured	1	2.0	
Pulse rate (b/min)			
Low (<70)	9	17.6	(8.9–31.4)
Normal (70–75)	6	11.8	(4.9–24.6)
High (>75)	35	68.6	(54.0–80.5)
Not measured	1	2.0	
Waist circumference (cm)			
Normal (M <102/F <88)	45	88.2	(75.4–95.1)
Overweight (M ≥102/F ≥88)	6	11.8	(4.9–24.6)
BMI (kg/m²)			
Underweight (<18.5)	1	2.0	(0.1–11.8)
Normal (18.5–24.9)	37	72.5	(58.0–83.7)
Overweight (>24.9)	13	25.5	(14.8–39.9)
ALT (U/L)			
Normal (M <41/N <31)	36	70.6	(56.0–82.1)
High (M ≥41/N ≥31)	15	29.4	(17.9–44.0)
AST (U/L)			
Normal (M <38/F <32)	26	51.0	(36.8–65.0)
High (M ≥38/F ≥32)	25	49.0	(35.0–63.2)
GGT (U/L)			
Low (M <10/F <5)	0	0.0	–
Normal (M 10–66/F 5–39)	35	68.6	(54.0–80.5)
High (M >66/F >39)	16	31.4	(19.5–46.0)
CDT (%)			
Low (<1.19)	6	11.8	(4.9–24.6)
Normal (1.19–2.47)	23	45.1	(31.4–59.5)
High (>2.47)	22	43.1	(29.6–57.7)
hsCRP (mg/L)			
Normal (<5)	30	58.8	(44.2–72.1)
High (≥5)	21	41.2	(27.9–55.8)
TChol (mmol/L)			
Normal (<5)	33	64.7	(50.0–77.2)
High (≥5)	18	35.3	(22.8–50.0)
HDL-Chol (mmol/L)			
Normal (>1)	51	100.0	–
High (≤1)	0	0.0	–
LDL-Chol (mmol/L)			
Normal (<3)	35	68.6	(54.0–80.5)
High (≥3)	15	29.4	(17.9–44.0)
TG (mmol/L)			
Normal (<1.7)	45	88.2	(75.4–95.1)
High (≥1.7)	6	11.8	(4.9–24.6)
HbA1c (%)			
Low (<4.8)	1	2.0	(0.1–11.8)
Normal (4.8–5.9)	45	88.2	(75.4–95.1)
High (>5.9)	5	9.8	(3.7–22.2)
ApoA1 (g/L)			
Low (M <1.0/F <1.1)	0	0.0	–
Normal (M 1.0–2.0/F 1.1–2.3)	43	84.3	(70.8–92.5)
High (M >2.0/F >2.3)	8	15.7	(7.5–29.1)
ApoB (g/L)			
Low (M <0.7/F <0.6)	18	35.3	(22.8–50.0)
Normal (M 0.7–1.3/F 0.6–1.2)	29	56.9	(42.3–70.4)
High (M >1.3/F >1.2)	4	7.8	(2.5–19.7)
Lp(a) (g/L)			
Normal (M <0.09/F <0.11)	29	56.9	(42.3–70.4)
High (M ≥0.09/N ≥0.11)	22	43.1	(29.6–57.7)
Gluc (mmol/L)			
Low (<4.1)	3	5.9	(1.5–17.2)
Normal (4.1–5.9)	28	54.9	(40.5–68.6)
High (>5.9)	20	39.2	(26.2–53.9)
TChol/HDL ratio			
Low (M <4.0/F <3.8)	46	90.2	(78.6–96.7)
Normal (M 4.0–23/F 3.8–11)	5	9.8	(3.3–21.4)
High (M >23/F >11)	0	0.0	–
LDL/HDL ratio			
Low (M <1.1/F <1.6)	14	27.5	(15.9–41.7)
Normal (M 1.1–7.9/F 1.6–6.0)	37	72.5	(58.3–84.1)
High (M >7.9/F >6.0)	0	0.0	–

SBP = systolic blood pressure; DBP = diastolic blood pressure; BMI = body mass index; ALT = alanine aminotransferase; AST = aspartate aminotransferase; GGT = gamma-glutamyltransferase; CDT =serum carbohydrate-deficient transferring; hsCRP = high-sensitivity C-reactive protein; TChol = total cholesterol; HDL-Chol = high-density lipoprotein cholesterol; LDL-Chol = low-density lipoprotein cholesterol; TG = triglycerides; HbA1c = glycated hemoglobin; ApoA1 = apolipoprotein A-1; ApoB = apolipoprotein B; Lp(a) = lipoprotein(a); Gluc = glucose.

Next, two ratios (TChol/HDL-Chol and LDL-Chol/HDL-Chol) are used worldwide for acute MI risk estimation in all ethnic groups, in both sexes and all ages (19). In our study group these ratios were also within the normal range (Table I).

ApoB is one of the principal proteins of the atherogenic lipoprotein particles (LDL, intermediate-density lipoprotein (IDL), very-low-density lipoprotein (VLDL)), and ApoA1 is a major protein component of HDL. A number of clinical studies demonstrate the association between low ApoA1 levels and an increased risk of MI and coronary artery disease. Also an increased ApoB/ApoA1 ratio has a role in clinical CVD development, including subclinical atherosclerosis (20). In addition, according to a large comprehensive study (INTERHEART study) the ApoB/ApoA1 ratio was superior for the estimation of the risk of acute myocardial infarction in all ethnic groups, in both sexes and all ages (19). Our study results showed a contradiction—no differences between average values of ApoA1 and ApoB compared to reference values were found. The percentage distribution demonstrated that 84.3% of persons had normal ApoA1, and 56.9% had normal ApoB value.

The values related to TChol and its fractions have been shown to be significantly influenced by life-style (physical activity, diet, etc.) and, through this, related to a higher risk of CVD development (21,22). Our study subjects, who were physically inactive and on unhealthy diets, had predominantly normal average TChol and HDL-Chol values. Over half of the subjects had normal LDL-Chol levels.

There is evidence that elevated levels of TG are associated with increased risk of MI and ischemic heart disease (23). Also it is known that systematic aerobic training-load reduces TG levels (24). In our study only six persons (11.8%) had elevated levels of TG. This phenomenon cannot be explained by systematic activity because the persons under investigation were in very bad physical condition. At the same time no relationships have been found between TG and chronic alcohol consumption (25). The effect of physical activity in our study is contradictory because, for on the one hand it is relatively necessary to move (walk) a lot to look for food from the trash bins, and on the other hand the subjects' very bad muscular and skeletal system (amputated toes, necrotic ulcers, complications after bone fractures, etc.) does not allow an intensive physical load. One explanation for this relatively low/normal TG level can be low-fat food consumption (which has not been investigated in this study).

CVD predictive markers (TChol, LDL-Chol, HDL-Chol, and TG) used in everyday clinical practice did not have alarming concerns among homeless people (more than half of the subjects had mentioned values within endemic norm; Table I). It seems that some other risk factors (ApoB and ApoA1) have also a certain limitation in their application.

What kind of risk markers/aspects should be taken into account when this kind of population is under investigation? It seems that inflammation-related markers are more informative.

Among the subjects, the mean hs CRP value was twice as high as the reference value (<5 mg/L), but percentage evaluation showed that 58.8% of investigated persons had normal values. This kind of distribution was influenced by two persons whose values were above100 mg/L. This kind of hsCRP elevation is obviously caused by chronic inflammations due to what subjects suffer without getting any cure or medication (legs wounds, pneumonia, dermatitis, etc.).

The risk of cardiovascular morbidity and mortality is greatly affected by cigarette smoking (26), and in our study 94% of participants were smokers. Smoking is claimed to be associated with a lower socio-economic status (27), and the prevalence of nicotine dependence among alcohol or other substance abusers is extremely high (28) and is well documented among homeless people (29–31). The measurement of CDT is the only test approved by the Food and Drug Administration (FDA) for determining heavy alcohol use (32). Our results for CDT% showed higher mean values of hepatic enzymes compared to reference values. Surprisingly 45.1% of investigated persons had normal values of CDT.

In the scientific literature, a faster resting pulse rate has been shown to be associated with a higher risk of developing hypertension and a greater incidence of cardiovascular morbidity and mortality (26,33).In our study 68.6% of investigated patients had elevated pulse rates. This can be explained by sympathetic over-activity, which is related to the following circumstances: smoking, alcohol intoxication, physical inactivity, depression, and poor self-rated health (34).

Considering that in the shelter there was no systematic eating and no opportunity to cook, the BMI of the investigated persons was normal compared to the reference value. Only one person (*c.* 2% of the research population) was underweight, and waist circumference indices were all at normal range or above. It can be concluded from this that the investigated group of homeless people did not suffer from starvation. An explanation can be found in recent research, which brought forth theinterconnection between long-term alcohol consumption and heightened BMI (35). Unfortunately, accurate nutrition research is methodically difficult to conduct, and also it was not the aim of the given research.

Persistent elevations in HbA1c level increase the risk for the long-term vascular complications of diabetes such as coronary disease, heart attack, stroke, heart failure, kidney failure, blindness, erectile dysfunction, neuropathy (loss of sensation, especially in the feet), gangrene, and gastroparesis (slowed emptying of the stomach). Elevated levels of HbA1c can also be associated with disorders of glucose metabolism, but among our investigated persons most levels (88.2%) were within normal range.

Of course our study had some limitations. Because experimentally it is unethical to recruit people to live under unhealthy conditions, the selection of the study contingent was made considering the objective of the study—to examine CVD markers in a social group living unsalubrious lives. The homeless people are not an ideal group of choice for the study, because the credible information of their prior life history (results of medical examination and medical records, etc.) is missing, and some results of the questionnaire were contradictory.

Based on life condition differentialities and social status profile between countries (climate, donations/foundations for the homeless) our participants do not constitute a representative sample of homeless people. Also, they were in a bad physical condition, which raises questions about the situation elsewhere. Based on that, generalizations cannot be made.

An ideal structure of the research would also be to assess the ‘amount’ of the unhealthy life-style choices, i.e. the quantity of alcohol and drugs consumed, smoking, physical capacity, etc. In this study, only objective results of the physical and laboratory analysis were used.

Originally it was planned to include a controlgroup, but to find people whose life-style was 100% objectively proven and correspondent to that of homeless people was difficult (not controllable). Hence, the outcome was compared with reference indices (analogously used in daily clinical practice). Confidence interval gives us the opportunity to generalize the research results to the whole homeless contingent.

## Conclusions

Our study demonstrates that traditional markers used for prediction and diagnosis and treatment of CVD may not demonstrate the same information pattern among people who live unsalubrious lives—homeless people. The fact is that CVD is still the leading cause of death among homeless people (9,10), and this kind of life-style cannot be understood to be healthy for the cardiovascular system, but it certainly throws light on the complexity and multifactorial etiology of CVD development mechanisms and may demonstrate the weak points of widely used diagnostic markers.
